# Pharmacological Induction of Fetal Hemoglobin in β-Thalassemia and Sickle Cell Disease: An Updated Perspective

**DOI:** 10.3390/ph15060753

**Published:** 2022-06-16

**Authors:** Rayan Bou-Fakhredin, Lucia De Franceschi, Irene Motta, Maria Domenica Cappellini, Ali T. Taher

**Affiliations:** 1 Department of Clinical Sciences and Community Health, University of Milan, 20122 Milan, Italy; rayan.boufakhredin@unimi.it (R.B.-F.); irene.motta@unimi.it (I.M.); 2 Department of Medicine, University of Verona and Azienda Ospedaliera Universitaria Verona, 37128 Verona, Italy; lucia.defranceschi@univr.it; 3 UOC General Medicine, Fondazione IRCCS Ca’ Granda Ospedale Maggiore Policlinico, 20122 Milan, Italy; 4 Department of Internal Medicine, Division of Hematology-Oncology, American University of Beirut Medical Center, Beirut 1107 2020, Lebanon

**Keywords:** β-thalassemia, sickle cell disease, fetal hemoglobin, globin gene, γ-globin, pharmacological induction

## Abstract

A significant amount of attention has recently been devoted to the mechanisms involved in hemoglobin (Hb) switching, as it has previously been established that the induction of fetal hemoglobin (HbF) production in significant amounts can reduce the severity of the clinical course in diseases such as β-thalassemia and sickle cell disease (SCD). While the induction of HbF using lentiviral and genome-editing strategies has been made possible, they present limitations. Meanwhile, progress in the use of pharmacologic agents for HbF induction and the identification of novel HbF-inducing strategies has been made possible as a result of a better understanding of γ-globin regulation. In this review, we will provide an update on all current pharmacological inducer agents of HbF in β-thalassemia and SCD in addition to the ongoing research into other novel, and potentially therapeutic, HbF-inducing agents.

## 1. Introduction

Hemoglobinopathies are among the most common inherited monogenic disorders in the world and they exhibit a large range of clinical phenotypes with varying disease severity [1,2,3]. The levels of fetal hemoglobin (HbF) in erythrocytes make up a large part of the clinical heterogeneity that is observed in patients with β-thalassemia and sickle cell disease (SCD).

HbF is the predominant form of Hb expressed throughout gestation and has a crucial role in facilitating transplacental gas exchange [4,5,6]. Composed of two α-globin chains and two γ-globin chains, HbF has a greater affinity for oxygen when compared to hemoglobin A (HbA) [4,7,8]. After birth, the predominant form of Hb produced switches from HbF to HbA, which has a lower affinity for oxygen [4,5,6,8,9,10]. This switch is genetically regulated by transcription factors such as BCL11A, KLF-1 and MYB [4,11] and results in adults typically expressing low levels of HbF [4,12,13]. However, as expression switches from HbF to HbA, individuals who have mutations in the β-globin gene end in either deficiency in globin chain synthesis or production of pathologic Hb such as in β-thalassemia or SCD. Fetal erythrocytes or fetal cells contain nearly 100% of HbF. Following the transcriptional switch in definitive erythroid progenitors from HbF (α2γ2) to adult hemoglobin (α2β2) around birth, very low levels of HbF are found a year after birth [14], suggesting this switch is not complete. In some individuals, higher levels of HbF persist into childhood and adulthood, reflected in a continuous positively skewed distribution of HbF across healthy adults. Adult erythrocytes that contain detectable levels of HbF are called F cells. These cells arise from erythroid precursors that can give rise to both HbF-containing and non-HbF-containing erythrocytes [15]. Hereditary persistence of fetal hemoglobin (HPHF) is defined as the heterogenous group of inherited defects in the switch from fetal to adult hemoglobin [16]. Large deletions or point mutations within the HBB gene cluster that result in higher than normal levels of HbF persisting into adulthood cause the most frequently recognized type of HPFH. Pancellular vs. heterocellular HbF distribution has been used as a defining aspect of HPFH [16]. The three main loci that control HbF levels outside of the locus control region at the 5’ end of the β-globin gene cluster and promoter regions on the β- and γ-globin genes are BCL11A, ZBTB7A and MYB [15].

**Figure 1:**
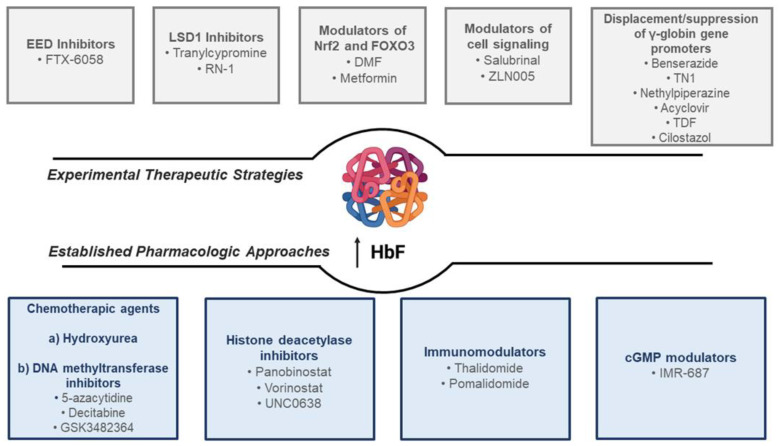
Summary of all established pharmacological approaches and experimental therapeutic strategies for HbF induction in β-thalassemia and SCD.

In recent years, progress on the knowledge of molecular mechanisms involved in Hb switching, the concept of HPHF, combined with epidemiological and clinical observations has provided important evidence on the beneficial role of increasing HbF levels in ameliorating the clinical complications of β-thalassemia and SCD [17]. This can be achieved by either classic drug-modulating approaches or by gene therapy and genome-editing approaches [18,19,20,21,22,23,24]. Although encouraging and promising results have been reported on gene therapy/genome editing and HbF expression, their safety is still to be determined and their expected costs are to be considered as well [25,26,27]. Accordingly, pharmacological induction of HbF production is still an interesting option to decrease the severity of these disorders. In this review, we will provide an update on all current pharmacological inducer agents of HbF in β-thalassemia and SCD in addition to the ongoing research into other novel and potentially curative HbF-inducing agents (Figure 1).

## 2. Established Pharmacologic Approaches to HbF Induction in Hemoglobinopathies

Different pharmacological agents have been pre-clinically and clinically tested at various stages of clinical trials for their ability to increase γ-globin expression. In this section, we describe these already established and major pharmacological approaches and agents for HbF induction. These are also summarized in Figure 1.

### 2.1. Chemotherapeutic Agents

#### 2.1.1. Hydroxyurea

The primary mechanism of action of hydroxyurea (HU) in β-thalassemia and SCD is the upregulation of the γ-globin gene expression in erythroid cells [28]. Hydroxyurea enhanced the percentage of F cells in the circulations of patients with hemoglobinopathies, with a heterogenous distribution within red cells, leading to overall improvements in the hematologic phenotype [29,30,31,32,33]. While the clinical efficacy of HU is primarily due to its ability to induce HbF, its exact mechanism of action remains not fully understood [34]. 

Studies conducted in β-thalassemia patients showed that HU induced a 2-to-9-fold increase in γ-mRNA with a good correlation between γ-mRNA and HbF levels [35,36,37,38]. Responses in these patients were observed at HU doses ranging between 10 and 20 mg/kg/day. In addition to its known effects in stimulating γ-globin production, the use of HU was also associated with a decrease in transfusion requirement and an improvement in the prothrombotic profile, especially in splenectomized patients [39,40]. Collectively, these studies present several limitations such as the small sample size, the definition of transfusion dependence, the heterogeneity of the β-thalassemic patient populations and the lack of control groups. Thus, we might conclude that these data generate a rationale to design new studies to assess the possible beneficial effects of HU in β-thalassemia patients, particularly in those with non-transfusion-dependent thalassemia.

Improvements in HbF levels after HU treatment have been shown in patients with SCD, HbSC disease, HbS-β^0^ and HbS-β^+^ thalassemia [40,41,42,43]. In SCD, two different mechanisms exist for HU-induced increases in HbF synthesis: (i) inhibition of ribonucleotide reductase, promoting the selection of high HbF expression erythroid precursors and (ii) the direct selection of HbF cell production by inhibiting soluble guanylate cyclase [44,45,46]. Several studies on SCD have proposed that curative therapies with HU should aim to achieve HbF >30%, F cells >70% and >4–10 pg F/F cell [47,48,49]. Other studies demonstrated that the early initiation of HU using individualized and pharmacokinetics-guided dosing can lead to robust and sustained HbF levels beyond 30–40% in most SCD patients who are adherent to therapy [50,51]. Hydroxyurea has also been associated with improved mortality and morbidity in both adults and children with SCD [52]. Indeed, HU significantly reduced hospitalization rate, acute chest syndrome, VOCs and transfusion requirements [53]. Few studies have shown a possible declining effect of HU on HbF synthesis over time [54]. The beneficial effects of HU are noteworthy and go beyond HbF synthesis in SCD. Hydroxyurea also acts as a multimodal agent targeting neutrophils, modulating inflammatory response and vascular endothelial activation, contributing to nitric oxide biosynthesis [55,56,57,58].

#### 2.1.2. DNA Methyltransferase Inhibitors

DNA methyltransferase (DNMT) inhibitors, such as 5-azacytidine and decitabine, can reactivate γ-globin gene expression via DNA hypomethylation [59]. Initially, 5-azacytidine was studied and used in SCD but it was quickly abandoned due to its toxicity profile and possible carcinogenicity risk [60]. Decitabine, an analogue of 5-azacytidine, is also a potent DNMT1 inhibitor with a more favorable safety profile. A pilot study by Oliviera et al. showed that decitabine administered subcutaneously at a dose of 0.2 mg/kg twice per week for 12 weeks increased total hemoglobin (Hb) from 78.8 to 90.4 g/L, and increased absolute HbF levels from 36.4 to 42.9 g/L in 5 β-TI patients [61]. An improvement in RBC indices was also noted. Treatment was well tolerated overall, with the main adverse event being an elevation in platelet counts [61]. Small studies in SCD have also suggested that decitabine can substantially increase HbF and total Hb levels [62,63,64,65]. However, if taken orally, decitabine is rapidly deaminated and inactivated by cytosine deaminase. To overcome this challenge, one clinical trial used decitabine in combination with tetrahydrouridine (THU), a cytosine deaminase inhibitor, to induce HbF production (NCT01685515). A phase I study showed that the combination of decitabine and THU led to the persistent inhibition of the DNMT1 protein with induction and increase in HbF levels, and more importantly, HbF-enriched red cells (F cells) increased to 80%. While these agents do not have major myelotoxic effects, they might induce thrombocytosis. This might be taken into consideration in patients with SCD that is per se a thrombophilic state [66].

Recently, an innovative and orally bioavailable DNMT1-selective inhibitor known as GSK3482364 has emerged [67]. In contrast with the cytidine analog DNMT1 inhibitors, the inhibitory mechanism of GSK3482364 does not require DNA incorporation and is reversible. In cells, treatment with GSK3482364 caused DNA hypomethylation, and resulted in HbF induction. These effects were approximately equivalent and comparable to decitabine treatment [67]. In an in vitro model of erythropoiesis, GSK3482364 and decitabine led to comparable increases in HbF cells. However, treatment with GSK3482364 resulted in a larger proportion of cells maturing into HbF-expressing reticulocytes [67]. The effects of GSK3482364 on the bone marrow of transgenic SCD mice were also studied and showed a clear induction of F cells that exceeded the corresponding effects of decitabine at tolerated doses over a 12-day period [67]. The compound was well-tolerated by SCD mice and there was no evidence of adverse hematological effects [67]. Notably, and unlike what has been reported and observed with decitabine, the use of GSK3482364 was not associated with significant increases in platelet count. Taken together, these data suggest that GSK3482364 is a promising molecule to further study the role of DNMT1 inhibitors in both in vitro and in vivo models of SCD.

### 2.2. Histone Deacetylase Inhibitors

Histone deacetylase (HDAC) inhibitors have also been considered as therapeutic targets for HbF induction. This group of regulatory molecules is involved in the epigenetic silencing of the γ-globin genes [34,68]. The earliest HDAC inhibitor investigated and used as a HbF inducer was butyrate. Butyrate increased the transcription rate of the HBG genes, as well as the translation of HBG1/HBG2 mRNA [69]. In clinical trials conducted on patients with β-thalassemia and SCD, intravenous administration of arginine butyrate and oral administration of sodium phenylbutyrate increased HbF levels. However, these oral butyrate compounds were only successful in producing a very mild raise in HbF levels and the overall patient compliance was poor [70]. Panobinostat is a pan-HDAC inhibitor that has been reported to have a greater potency than sodium butyrate [71]. It is currently being tested in a phase I clinical trial on adult SCD patients (NCT01245179). 

Vorinostat (also known as suberoylanilide hydroxamic acid or SAHA) is a hydroxamic acid group pan-HDAC inhibitor that has been used in many research studies including cancer treatment [72]. In 2019, Mettananda et al. demonstrated that in human erythroid cells, vorinostat downregulates α-globin expression while inducing γ-globin expression and its use can thus be considered as a potential therapy for β-thalassemia [73]. As a potent inducer of γ-globin and HbF, vorinostat has also been considered as a potential therapy for SCD [74]. Thus, it was postulated that vorinostat may help in the treatment SCD by increasing the amount of HbF in the blood. One 2010 study by Hebbel et al. showed that the HDAC inhibitors trichostatin A and vorinostat were beneficial for the vascular pathobiology of sickle transgenic mice and led to an increase in HbF levels [75]. A phase 2 trial assessing the safety and efficacy of vorinostat (once a day for three consecutive days every week for 12–16 weeks) in adult SCD patients resistant to HU was also initiated (NCT01000155). However, the study was eventually terminated early as recruitment was poor. A novel class I-restricted HDAC inhibitor and largazole analog known as CT-101 has been recently evaluated for its pharmacodynamics, cytotoxicity and targeted epigenetic effects in sickle erythroid precursors [76]. Results demonstrated that CT-101 was successfully able to activate γ-globin transcription selectively and increase F cells and HbF levels [76]. Moreover, the combination of CT-101 with HU produced a better effect on HbF levels. CT-101 produced very limited cell toxicity after 5 days of treatment as shown by slightly reduced sickle erythroid maturation. Despite this, cell numbers continued to increase [76]. CT-101 also increased acetylated histone H3 and H4 levels and conferred an open chromatin conformation in the γ-globin promoter [76]. Thus, CT-101 may be a possible lead candidate to be further developed and transferred to clinical studies as a pharmacologic inducer of HbF.

#### EHMT1/2 Inhibitors

Other pharmacological agents targeting histone methyl transferase to induce HbF expression include euchromatic histone-lysine-*N*-methyltransferases 1 and 2 (EHMT1/2) inhibitors. In vitro, UNC0638 has been shown to induce γ-globin mRNA and HbF expression in normal erythroid precursors [77,78,79]. This was associated with a decreased accumulation of H3K9me2 near the γ-globin loci and with increased loop formation between the locus control region and the γ-globin promoters through the recruitment of the LDB1 complex to the γ globin promoters [77,78,79]. In a recent study conducted on erythroid precursors derived from CD34+ cells of β^0^-thalassemia/HbE patients, the HbF-inducing activity of UNC0638, either alone or in combination with pomalidomide and decitabine, was investigated [80]. UNC0638 was able to increase in γ-globin mRNA levels, HbF levels and F cells. This HbF induction was 25.5 ± 4.2% above baseline levels, with a more pronounced effect when added at an early stage (day 4) of erythropoiesis [80]. It is noteworthy that UNC0638 exhibited an HbF-inducing activity similar to pomalidomide, but higher than decitabine under the same culture conditions [80]. UNC0638 shows a synergic effect on HbF synthesis when used in combination with either pomalidomide or decitabine. Although UNC0638 showed a strong HbF-inducing activity in ex vivo erythroid cell culture systems, its in vivo pharmacokinetic properties were shown to be due to a lack of drug-like properties [81]. In the future, EHMT1/2 inhibitors should undergo further development to achieve a higher potency and improved in vivo pharmacokinetic properties.

### 2.3. Immunomodulators: Thalidomide and Its Derivatives 

Thalidomide is an immunomodulatory compound used in clinical practice for the clinical management of multiple myeloma [82,83]. Thalidomide and its derivate pomalidomide have been considered as new therapeutic options for β-thalassemia. In vitro models of pathologic erythropoiesis have established that thalidomide might induce γ-globin mRNA expression in a dose-dependent manner throughout the modulation of γ-globin mRNA expression targeting BCL11A, SOX6, GATA1 and KLF1 as well as by post-translational modification induced by p38MAPK activation [82,83,84]. Several small case series and few observational studies have reported the efficacy and safety of thalidomide in patients with β-thalassemia [85,86,87,88,89,90,91,92]. Collectively, these studies show that thalidomide (range dosage from 75–100 mg/kg/day to 150–200 mg/kg/day) increases Hb levels by elevating the HbF level and reduces spleen size [93,94,95,96,97]. Furthermore, the efficacy of thalidomide on hematologic phenotype of patients with β-thalassemia was not inferior of HU [93]. Since large doses of thalidomide can be detrimental, the lowest effective dose of 50 mg/day was proposed to improve anemia in β-thalassemic patients [93,94,98,99]. In the future, larger randomized controlled trials are needed to establish the efficacy of thalidomide in patients with β-thalassemia. 

Pomalidomide, a third-generation immunomodulatory drug and thalidomide derivative with less adverse events (AEs), has been shown to be an effective and potent HbF inducer in in vitro β-thalassemia/HbE erythropoiesis [94]. Its use has also been documented to increase HbF levels in SCD. Similar to HU, pomalidomide increased the level of HbF production without myelosuppressive effects in a humanized mouse model of SCD [100]. In addition, treatment of human hematopoietic stem cells (HSCs) with pomalidomide and lenalidomide significantly stimulated the proliferation of HSCs and induced HbF by modulating the transcription of HBB and HBG gene through the downregulation of BCL11A IKZF1, KLF1, LSD1 and SOX-6 repression factors [82,101]. A phase 1 trial was conducted to assess the efficacy, safety and maximum tolerated dose of pomalidomide (0.5–4 mg/day for 84 days) in SCD patients (NCT01522547). Two out of four patients treated with pomalidomide at 4 mg/day showed an increase in HbF levels [15]. Lately, pomalidomide–nitric oxide donor derivatives (3a–f) have been synthesized and their suitability as novel and potential HbF inducers has also been evaluated and successfully shown in an early preliminary study [70]. This could pave the way in the future for further investigation to use these pomalidomide derivatives in the treatment of SCD.

### 2.4. cGMP Modulators: Phosphodiesterase-9 Inhibitor 

IMR-687 (Tovinontrine) is a highly selective phosphodiesterase 9 inhibitor that is currently being developed as an orally administered therapy for β-thalassemia and SCD patients. Similar to HU, IMR-687 increases intracellular cGMP levels and its use in preclinical studies showed an increase in HbF expression [102]. In a phase 2a study conducted on adult patients with SCD (*N* = 93) (NCT03401112), IMR-687 was given at a dose of 50–200 mg once daily (*N* = 63) for up to 6 months. IMR-687 was generally well-tolerated when used alone or in combination HU and the data showed a mean absolute change of +1.9 and +7.3 in HbF (%) and F cells (%) at 4 months, respectively, with minimal changes in Hb [103]. A phase 2a open-label extension study (NCT04053803) is ongoing to assess the long-term efficacy and safety of IMR-687 in SCD patients for up to 4 years at a dose of 200 mg once daily [104]. Seventeen patients with SCD were treated with IMR-687 monotherapy and seven were treated with a combination of IMR-687 and HU. Preliminary results from the patients that had an evaluable pharmacodynamic data at 8 months showed that seven (47%) patients had a ≥6% absolute increase in F cells and four patients (36%) had a ≥3% absolute increase in HbF [104]. With these encouraging data, a phase 2b study in SCD patients is currently ongoing to further evaluate the efficacy of IMR-687 as a HbF-inducing agent at higher doses (NCT04474314).

A summary of the mechanism of action of these established pharmacologic approaches to HbF induction has been included in Table 1. 

## 3. Novel Experimental Strategies for the Pharmacologic Induction of HbF in Hemoglobinopathies

Progress on the knowledge of molecular mechanisms involved in the modulation of HbF synthesis has paved the way for the identification of new potential therapeutic targets, which are currently under evaluation at the pre-clinical stage to then possibly be transferable to clinical studies in patients with in hemoglobinopathies (Figure 1). 

### 3.1. FTX-6058: Embryonic Ectoderm Development (EED) Inhibitor

FTX-6058 is a small and potent molecule that acts as an inhibitor of embryonic ectoderm development (EED), which is part of the polycomb repressive complex 2 (PRC2) that is involved in the repression of gene transcription by histone 3 methylation. In both in vitro and in vivo models of SCD, FTX-6058 induced the upregulation of HbF expression (up to 40% total Hb) [105]. The safety and efficacy of single- and multiple-ascending doses of FTX-6058 are currently being investigated in a phase-1 trial enrolling healthy adult volunteers (NCT04586985) [106]. FTX-6058 was tested at the dosage of 2 mg to 60 mg both given once daily for 14 days vs. placebo [106]. Interim results showed proportional increases in HbF levels, and increased numbers of F reticulocytes in the dose-ascending groups [106]. After 14 days, the 6 mg dose increased mRNA levels by more than three-fold and the 10 mg dose by more than four-fold. New data from the group of patients receiving a dose of 20 mg and 30 mg showed that by day 14, Hb mRNA levels had risen by a mean of 5.6 times and 6.2 times, respectively [106]. Moreover, 7 to 10 days after dosing, the percentage of F reticulocytes increased by a mean of 1.8 times and 2.4 times in the 20 mg group and 30 mg group, respectively [106]. FTX-6058 was generally well-tolerated without major AEs reported. All the above mentioned results further support the rationale to design clinical studies with FTX-6058 in patients with SCD. Indeed, a phase 1b proof-of-concept study is awaited to evaluate FTX-6058 in people with SCD [106]. 

### 3.2. Lysine-Specific Histone Demethylase 1 (LSD1) Inhibitors

With the use of RNA interference strategies and pharmacological inhibitors, lysine-specific histone demethylase 1 (LSD1) has been identified as a therapeutic target for HbF induction [107]. Tranylcypromine (TC) is a selective monoamine oxidase inhibitor of LSD1 that is currently FDA approved and used for the management of major depressive disorders [108]. Shi et al. initially reported that inhibition of LSD1 by TC enhances HbF synthesis in primary human erythroid cells, as well as in β-YAC mice [107]. However, in vitro erythropoiesis, high TC concentrations delayed erythroid maturation with a rapid decrease in total β-like globin mRNA [109]. 

Additionally, RN-1 is a TC analog that can act as an effective, irreversible LSD1 inhibitor with IC50 lower than TC (0.07 µM vs. 2 μM) [110]. In humanized healthy and SCD mice, LSD1 inactivation by RN-1 was shown to induce γ-globin expression and HbF synthesis [111]. In particular, treatment with RN-1 increased the expression of murine embryonic εy- and βh1-globin genes without any changes in the expression of adult β-globin [111]. When tranylcypromine (TCP) and RN-1 were tested in primate and murine erythroid cell cultures, RN-1 was shown to induce F cells and γ-globin mRNA at much greater levels than either TCP or HU [112]. More recently, the use of RN-1 was investigated in erythroid progenitor cells derived from β0-thalassemia/HbE patients [113]. At a concentration of 0.004 uM, RN-1 significantly increased the expression of γ-globin mRNA and HbF expression without any significant toxicity and regardless of HbF baseline levels [113]. Transcript levels of numerous key γ-globin repressors and co-repressors such as NCOR1, SOX6 and MYB were also modulated by RN-1 treatment [113]. Collectively, these data suggest the consideration of RN-1 for further development as an additional strategy to induce HbF in both SCD and thalassemic syndromes.

### 3.3. Modulators of Redox-Related Transcriptional Factors: Nrf2 or FOXO3 

#### 3.3.1. Dimethyl Fumarate

Dimethyl fumarate (DMF), a small and orally active molecule that acts as a nuclear factor erythroid derived-2-like 2 (Nrf2) agonist, is currently approved for the treatment of relapsing-remitting multiple sclerosis [114,115,116,117]. Being a transcription factor, Nrf2 triggers the cytoprotective and antioxidant pathways in response to oxidation [118,119,120,121]. A study by Krishnamoorthy et al. examined the ability of DMF to activate γ-globin transcription and enhance HbF in SCD-derived erythroid precursors cells, SCD transgenic mice and non-anemic cynomolgus monkeys [122,123]. Across all these settings, DMF (with or without HU) significantly increased the level of γ-globin mRNA, the ratio of γ/β-globin mRNA and the percentage of F cells [122]. The greatest average increase in γ/β-globin mRNA was observed in the group receiving a combination of DMF and HU [122]. This was associated with increased ^G^γ-globin and ^A^γ-globin chains synthesis [122]. In humanized SCD, DMF (100 mg/kg) enhanced ^A^γ-globin expression and Nrf2-dependent genes such as NQO1 and HO-1 with beneficial effects in murine hematologic sickle cell phenotype as well as an improvement in inflammatory vasculopathy [122]. These findings are in agreement with previously published studies where Nrf2 was genetically activated by decreasing expression of one its binding protein Keap1 [124,125]. Thus, DMF might be an interesting new approach to the biocomplexity of SCD by acting as multimodal therapy, not only as an HbF inducer agent but also as a modulator in inflammatory response and vascular dysfunction. 

#### 3.3.2. Metformin

Metformin has been shown to activate the redox-related transcriptional factor FOXO3 in both non-erythroid cell lines and hepatocytes [126,127,128]. Gene silencing of FOXO3 reduced γ-globin RNA expression and HbF levels in erythroblasts, whereas overexpression of FOXO3 produced the opposite effect [129]. When primary CD34+ cell-derived erythroid cultures were treated with metformin (0, 50 and 100 mM), dose-related FOXO3-dependent increases in the percentage of HbF as well as in the amounts of HbF-immunostaining cells (F cells) were observed, without any changes in BCL11A, MYB or KLF1 expression [129]. Studies in erythroid precursors from SCD patients treated with metformin alone or in combination with HU show a three-fold reduction in in vitro sickling, comparable to that observed with HU alone. It is noteworthy that the combination of metformin and HU have a synergic effect on the reduction in the percentage of sickled erythroid cells compared with monotherapy. Metformin was also evaluated in 18 patients with SCD with SS, Sβ^0^, Sβ^+^ and SC genotypes [130]. The increase in HbF was shown to be minimal in patients with SS, Sβ^0^ genotype [130]. Although some concerns on the use of metformin in SCD patients was raised due to the possible lactic acidosis which might accelerate HbS polymerization, no data on lactic acidosis were reported in patients with SCD [130]. Although the data on metformin are stimulating, the small number of SCD patients and the absence of data on clinical outcomes limit the conclusion of its use as a HbF inducer in clinical practice [131].

### 3.4. Agents Involved in Displacement/Suppression of γ-Globin Gene Promoters 

This family includes a heterogenous group of molecules, such as benserazide, TN1, nethylpiperazine, acyclovir, tenofovir disoproxil fumarate and cilostazol, which are involved in the displacement/suppression of γ-globin gene promoters such as LSD-1, BCL11A and HDAC3 [132] (Figure 1).

#### 3.4.1. Benserazide

Benserazide is a peripheral dopa decarboxylase inhibitor used in combination with L-DOPA for treatment of Parkinson’s disease. In preclinical models, benserazide was shown to induce HbF production [133,134]. Benserazide was also shown to increase fetal γ-globin gene transcription and the proportions of cells expressing HbF (F reticulocytes and F cells) in erythroid precursors from patients with HbE-β^0^-thalassemia and SCD [133,135]. An observational study by Santos et al. on a total of 50 individuals was conducted to evaluate the ability of benzeraside (at daily doses that ranged from 100 mg to 700 mg) to increase HbF production and circulating F cells. No correlations were found between the average daily dose of benserazide and HbF levels [136]. Moreover, no hematologic AEs related to benserazide use were recorded, even after up to 22 years of treatment [136]. Another recent study evaluated the efficacy of (R,S)-benserazide in comparison to its enantiomers to identify the best optimal form for clinical development transferable to hemoglobinopathies such as β-thalassemia or SCD [132]. Non-inferiority data on HbF expression between benserazide and its individual enantiomers were reported [132]. In addition, in β-YAC mice, the intermittent treatment with all forms of benserazide significantly increased the proportions of F cells with similar pharmacokinetic profiles in the absence of myelotoxicity [132]. Thus, the use of benserazide either alone or in combination with other HbF-inducing agents, such as HU or decitabine, might be considered another possible strategy to further increase HbF levels and proportions of F cells through complimentary mechanisms.

#### 3.4.2. Purine-Based Fetal Hemoglobin Inducers

An early study by Nam et al. provided the first evidence on TN1 (2,6-diamino-substituted purine), a potent purine-based HbF inducer [137]. In leukemia cell lines, TN1 (100 nM) induced HbF more potently than HU [137]. However, another study conducted on human primary erythroid cells showed that this agent did not significantly increase γ-globin gene expression [138]. These drawbacks limited the development of this compound and its capability of being used in clinical trials in patients with hemoglobinopathies. Lai et al. then reported another potent and orally active purine-based HbF inducer known as Nethylpiperazine, or compound **13a** [138]. In vitro assays demonstrated that in primary erythroid cells as well as in HU-resistant primary erythroid cells, compound **13a** might efficiently induce γ-globin expression at a non-toxic concentration [138]. In a mouse model for SCD, compound **13a** was safe and well tolerated with a dose-dependent beneficial effect on the hematologic SCD phenotype [138]. Thus, compound **13a** can be considered an interesting molecule to be further developed as an inducer of HbF. It may be used in combination with HU or in HU low-responder patients with β-thalassemia and SCD.

Acyclovir (ACV) is another cyclic purine nucleotide analog, recently reported to induce HbF. Acyclovir is an FDA-approved antiviral agent against herpes simplex viruses, and its antiviral activity is highly specific [139]. Ali et al. reported the increased expression of γ-globin gene and HbF synthesis in CD34+-derived erythroid precursors. No major effects on either erythroid proliferation and maturation were observed [140]. ACV significantly downregulated the γ-globin repressors BCL11A and SOX6. This was associated with the upregulation of GATA-1 [140]. Data from β-YAC transgenic mice treated with ACX revealed a substantial increase in HbF mRNA expression as well as in the percentage of F cells [140]. 

Tenofovir disoproxil fumarate (TDF) is an acyclic nucleotide analogue of adenosine used in the treatment of the human immunodeficiency virus and hepatitis B infection. TDF has also been investigated as a pharmacologically active HbF inducer. A study by Khan et al. observed that TDF increased erythroid differentiation, γ- globin gene mRNA transcription and HbF expression in K562 cells [141]. In vivo studies using β-YAC transgenic mice confirmed that the beneficial effects of TDF (at a dose of 200 mg/kg/day for four weeks) was even better than HU on the percentage of HbF-positive RBCs in the absence of myelotoxic and cytotoxic effects [141]. 

#### 3.4.3. Cilostazol (OPC-13013)

Cilostazol, a quinolinone derivative, is a reversible specific type 3 phosphodiesterase inhibitor. Cilostazol was shown to increase cyclic adenosine monophosphate (cAMP) cellular content, resulting in the inhibition of platelet aggregation and modulation of vascular tone [142]. Ali et al. demonstrated that cilostazol (20, 30, 40, 50 and 100 μM) induces erythroid differentiation of K562 cells in a concentration-dependent manner and significantly increases cell hemoglobinization [143]. This was associated with the upregulation of γ-globin mRNA transcripts as well as the amount of F cells [143]. In β-YAC mice, cilostazol upregulated γ-globin mRNA level and increased the percentage of F cells [143]. No adverse events secondary to the drug were observed. Although these are preliminary data, the multimodal action of cilostazol targeting vascular endothelial cells, platelets and HbF expression make this molecule an ideal candidate to further study in β-thalassemia and SCD. 

### 3.5. Molecules Targeting Post-Translational Modifications Involved in HbF Expression

Recent reports have highlighted the novel role of post-translational modifications such as phosphorylation in the induction of HbF, most likely amplifying signaling pathways activated in response to stress erythropoiesis [144,145] (Figure 1). 

#### 3.5.1. Salubrinal (SAL)

Salubrinal is a selective inhibitor of protein phosphatase 1 (PP1) that participates in the recruitment of phosphorylated eukaryotic initiation factor 2α (p-eIF2α) which in turn activates downstream targets such as activating transcription factor 4 (ATF4) [146]. Chen et al. demonstrated that SAL ameliorates anemia in mice genetically lacking the discoidal domain receptor1 (DDRGK1F/F), a model of stress erythropoiesis. [147]. Hahn et al. on the other hand showed that in normal erythroid precursors, SAL might prevent dephosphorylation of *p*-eIF2α, resulting in increased HbF production by a post-transcriptional mechanism [144]. Lopez et al. investigated whether SAL can induce HbF expression through the stress-signaling pathway by the activation of *p*-eIF2α and ATF4 trans-activation in the γ-globin gene promoter [148]. In sickle erythroid precursors, SAL (24 μM) increased F cells and significantly reduced oxidative stress, and increased levels of *p*-eIF2α and ATF4 [148]. In humanized SCD mice, a single intraperitoneal injection of SAL (at a dose of 5 mg/kg for four weeks) mediated a 2.3-fold increase in F cells and reduced the percentage of sickle erythrocytes and erythrocyte ROS production [148]. Although the study did not report an additive effect when SAL was used in combination with HU, it was concluded that SAL was as effective as HU[148]. 

#### 3.5.2. PGC-1α Agonist: ZLN005

The pharmacologic induction of peroxisome proliferator-activated receptor-γ coactivator 1-α (PGC-1α) has recently been shown to induce HbF gene expression [149]. PGC-1α interacts with different nuclear receptors such as PPARg or TR4, as well as in the recruitment of chromatin complexes. In human CD34+ cell-derived primary erythroid precursors, Sun et al. recently showed that the upregulation of PGC-1α by ZLN005, a small-molecule PGC-1α agonist, induced both γ-globin mRNA expression and HbF and increased F cells without significantly affecting cell proliferation and differentiation [149]. It was also reported that the use of ZLN005 in combination with HU exhibited an additive effect on the expression of γ-globin and the generation of F cells [149]. In addition, ZLN005 induced human γ-globin gene expression in SCD mice [149].

The mechanism of action of the abovementioned novel experimental strategies outlined in this section has also been summarized in Table 2.

## 4. Conclusions and Future Perspectives 

In conclusion, pharmacological agents to induce HbF are currently being used to alleviate the morbidity profile and disease burden that is associated with β-thalassemia and SCD. Understanding the complex regulation of HbF is key and necessary to supporting the generation of newer modalities. Research into novel pharmacologic strategies directed at elevating HbF levels is currently ongoing. The development of such novel curative treatments and approaches for β-thalassemia and SCD is crucial to diminish the clinical disease severity and substantially improve the quality of patients’ lives.

## Figures and Tables

**Table 1:** Mechanism of action of established pharmacologic approaches to HbF induction.

Agent	Mechanism of Action
**Hydroxyurea**	Ribonucleotide reductase inhibitor (inihibition of DNA analysis)
**DNA methyltransferase inhibitors**	Hypomethaltion of DNA and post-transcriptional mechanism
**HDAC inhibitors**	Inhibition of HDAC activity Epigenetic silencing of γ-globin genes
**Immunomodulators: Thalidomide** **and its derivatives**	Activation of p38 MAPK kinase Histone acetalation at γ-globin gene promoter
**cGMP modulators: PDE-9 inhibitor**	Inhibition of PDE9 and increased levels of cGMP

Abbreviations: HbF: Hemoglobin F; DNA: Deoxyribonucleic acid; HDAC: Histone deacetylase; cGMP: Cyclic guanosine monophosphate; PDE-9: Phosphodiesterase 9.

**Table 2:** Mechanism of action of novel experimental strategies for HbF induction.

Agent	Mechanism of Action
**EED inhibitors**	Inhibition of the activity of PRC2 (which catalyzes tri methylation of histone H3 at lysine 27) and elevation in gene expression (e.g., HBG1/2)
**LSD1 inhibitors**	Disruption of the DRED complex that controls the expression of γ-globin
**Modulators of redox-related transcriptional factors**	Activation of Nrf2 transcriptional pathway Enhancement of FOXO3 expression
**Agents involved in the displacement/ suppression of γ-globin gene promoters**	Displacement/suppression of γ-globin gene promoters
**Modulators of cell signaling**	Activation of *p*-eIF2α and ATF4 trans-activation in the γ-globin gene promoter Induction of PGC-1α

Abbreviations: HbF: Hemoglobin F; EED: Embryonic ectoderm development; PRC2: Polycomb repressive complex 2; Nrf2: Nuclear factor erythroid 2-related factor 2; FOXO3: Forkhead box O-3; p-eIF2α: Phosphorylated eukaryotic initiation factor 2α; ATF4: Activating transcription factor 4; PGC-1α: Peroxisome proliferator-activated receptor-γ coactivator 1-α.

## Data Availability

Not applicable.
